# Supplementary material for: Plasmodium falciparum translational machinery condones polyadenosine repeats
Source: eLife. 2020 May 29;9:e57799. doi: 10.7554/eLife.57799 (PMC7295572; doi:10.7554/eLife.57799)
Supplement: Supplementary file 1. — Background counts (Bgd count) represent all genes in defined GO group, result count are all genes with polyA tract found in identified GO group, percent of background (Pct of bgd) represents percent of polyA track genes in GO group. Fold enrichment is calculated over all polyA track genes as well as odd ratio. P-value as well as Benjamini and Bonferroni test values indicate statistical significance of GO analyses. [file elife-57799-supp1.rtf]

Supplementary File 1.
ID	Name	Bgd count	Result count	Pct of bgd	Fold enrichment	Odds ratio	P-value	Benjamini	Bonferroni	
GO:0016337	single organismal cell-cell adhesion	57	56	98,2	1,56	33,76	8,98E-11	6,69E-08	1,34E-07	
GO:0098602	single organism cell adhesion	57	56	98,2	1,56	33,76	8,98E-11	6,69E-08	1,34E-07	
GO:0007155	cell adhesion	77	71	92,2	1,47	7,15	2,66E-09	1,32E-06	3,97E-06	
GO:0009405	pathogenesis	101	81	80,2	1,28	2,44	0,00010834	0,04035608	0,16142433	
