# Supplementary material for: Plasmodium falciparum translational machinery condones polyadenosine repeats
Source: eLife. 2020 May 29;9:e57799. doi: 10.7554/eLife.57799 (PMC7295572; doi:10.7554/eLife.57799)
Supplement: Supplementary file 2. — P. falciparum genes related to human counterparts are given in brackets. [file elife-57799-supp2.rtf]

Supplementary File 2.
	Homo sapiens	Plasmodium falciparum	
No-Go Decay/Non- Stop Decay	Pelota	+	+    (PF3D7_0722100)	
	HBS1L	+	-	
	RACK1	+	+     (PF3D7_0826700)	
	XRN1	+	+    (PF3D7_0909400)	
	N4BP2 (Cue2, Nonu-1)	+	-	
Ribosome Quality Control	CNOT4	+	+    (PF3D7_1235300) 	
	ABCE1	+	+    (PF3D7_1368200)	
	ZNF598	+	+    (PF3D7_1450400) 	
	ASCC3 (Slh1)	+	+    (PF3D7_1439100)	
	NEMF	+	+    (PF3D7_1202600)	
	Listerin	+	+    (PF3D7_0615600)	
	UBE2D1	+	+     (PF3D7_1203900)	
